# Optic neuritis after ocular trauma in anti‐aquaporin‐4 antibody‐positive neuromyelitis optica spectrum disorder

**DOI:** 10.1002/brb3.2083

**Published:** 2021-02-16

**Authors:** Tetsuya Akaishi, Noriko Himori, Takayuki Takeshita, Kazuo Fujihara, Tatsuro Misu, Toshiyuki Takahashi, Juichi Fujimori, Tadashi Ishii, Masashi Aoki, Toru Nakazawa, Ichiro Nakashima

**Affiliations:** ^1^ Department of Neurology Tohoku University Graduate School of Medicine Sendai Japan; ^2^ Department of Education and Support for Regional Medicine Tohoku University Hospital Sendai Japan; ^3^ Department of Ophthalmology Tohoku University Graduate School of Medicine Sendai Japan; ^4^ Department of Multiple Sclerosis Therapeutics Fukushima Medical University Fukushima Japan; ^5^ Department of Neurology National Hospital Organization Yonezawa National Hospital Yonezawa Japan; ^6^ Department of Neurology Tohoku Medical and Pharmaceutical University Sendai Japan

**Keywords:** anti‐aquaporin‐4 antibodies, neuromyelitis optica spectrum disorders, ocular trauma, optic neuritis

## Abstract

**Objective:**

The aim of this study was to report the possible association between minor trauma to the eyes and the subsequent occurrence of optic neuritis in patients with serum anti‐aquaporin‐4 (AQP4) antibody‐positive neuromyelitis optica spectrum disorder (NMOSD).

**Methods:**

Herein, we present three patients who developed acute optic neuritis with visual disturbances after accidental minor trauma to their eyes, without any fundus abnormality or orbital floor fractures present.

**Results:**

Two of the three patients had a preceding history of neurological disturbances compatible with NMOSD (e.g., myelitis, area postrema syndrome) before the occurrence of trauma. One patient was rapidly treated with steroid pulse therapy and plasmapheresis, and he fully recovered visual acuity. The other two, who were left untreated in the acute phase, had sequelae of severe visual disturbances in the affected eyes.

**Conclusions:**

These cases suggest possible association between minor trauma to the eyes and the subsequent occurrence of optic neuritis in patients with serum anti‐AQP4 antibodies. Avoiding ocular trauma and early administration of steroid pulse therapy in response to optic neuritis after trauma are desired in such cases.

## Graphical abstract

We present three cases with serum AQP4‐IgG who developed optic neuritis after minor traumas. All patients showed relapsing clinical course compatible with AQP4‐IgG‐positive NMOSD. One patient treated with steroid pulse therapy had full recovery, but other untreated patients did not.

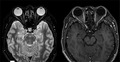

## 1 INTRODUCTION

Neuromyelitis optica spectrum disorder (NMOSD) is an autoimmune‐related neurological disease characterized by the presence of serum anti‐aquaporin‐4 autoantibodies (AQP4‐IgG) (Lennon et al., 2005). The clinical course of AQP4‐IgG‐positive NMOSD is characterized by recurrent episodes of acute optic neuritis (ON), acute myelitis, or area postrema syndromes (APS) (Wingerchuk et al., 2015). Given that the neurological disability in AQP4‐IgG NMOSD is known to almost exclusively after clinical attacks, presenting a stepwise disability progression pattern (Akaishi, Takahashi, Misu, et al., 2020; Weinshenker et al., 2015), both timely initiation of acute treatments to alleviate the subsequent irreversible neurological sequelae and relapse prevention treatments in the chronic phase are highly important. Although the presence of AQP4‐IgG in the serum has great significance in the diagnostic process and selection in therapeutic strategy, the exact pathophysiological mechanisms of this disease are still unclear. Several risk factors and possible triggers for neurological attacks, including traumatic injury, have been proposed recently. Eskandarieh et al. reported that a history of head trauma was a possible risk factor for the development of NMOSD based on the findings of their case–control study with an estimated crude odds ratio of 7.68 (95% confidence interval: 4.71–12.52) (Eskandarieh et al., 2018). Later, we reported that seven of the 53 patients with AQP4‐IgG‐positive NMOSD in our cohort had a recent history of traumatic injury or surgical operation in the month preceding the occurrence of attacks with neurological manifestations (Akaishi, Takahashi, Fujihara, et al., 2020). These reports support the hypothesis that physical trauma may play a triggering role in the clinical onset of NMOSD. In this case series, we discuss the clinical course of three patients with serum AQP4‐IgG who experienced ON episodes following a traumatic injury to their eyes.

## 2 METHODS

### 2.1 Study population

The present three cases are among the 86 patients with AQP4‐IgG‐positive NMOSD who were diagnosed and treated in our facility between 1990 and 2020. In the total cohort of 86 patients, the follow‐up period was 808 person‐years. During the follow‐up period, 137 episodes of acute ON were confirmed. The present three post‐traumatic acute ON episodes from three patients were extracted from these 137 episodes with acute ON from 57 patients (2.2% of the total ON episodes from 5.3% of the AQP4‐IgG‐positive patients with ON episodes).

### 2.2 AQP4‐IgG serology test

For the antibody testing, we conducted microscopic live cell‐based assay by using human M23‐AQP4‐expressing HEK293 cells, as we previously reported (Takahashi et al., 2006, 2007). Briefly, the HEK293 cells were incubated with 1:16 diluted serum samples, followed by staining with Alexa 488‐conjugated secondary antibody. The titers were calculated semi‐quantitatively using consecutive twofold end‐point dilutions.

### 2.3 Ethics

Measurement of serum AQP4‐IgG titer in the patients was approved by the Institutional Review Board of the Tohoku University School of Medicine. All study procedures were performed in accordance with the current version of the Declaration of Helsinki. Written informed consents for serum antibody testing were obtained from the patients. All three patients agreed to the publication of their anonymized clinical data.

## 3 RESULTS

**FIGURE 1:**
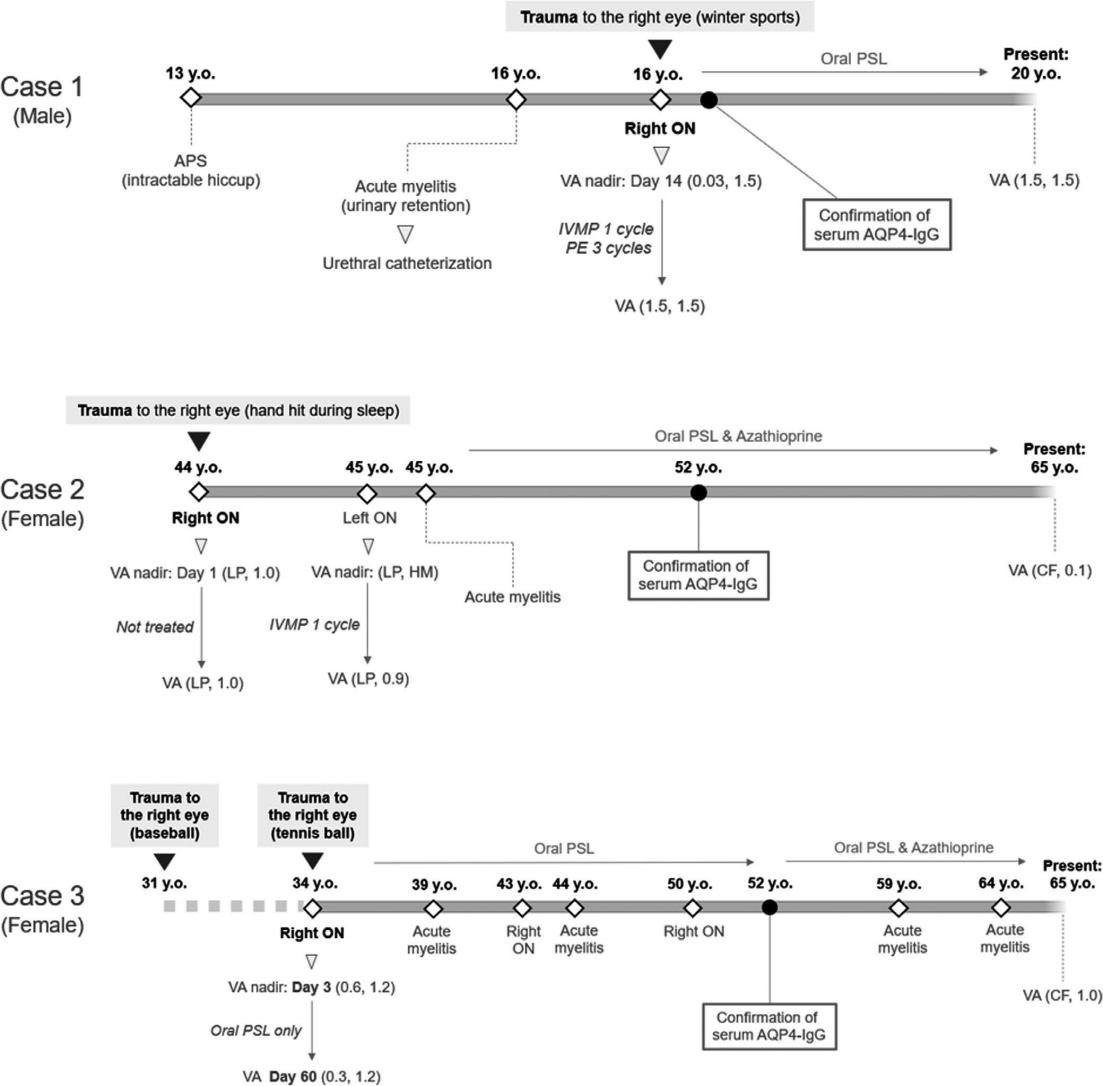
Clinical course of the present three cases with AQP4‐IgG‐related optic neuritis after minor traumas. All listed visual acuities (VA) are the corrected scores on the decimal scale. The white diamonds show the occurrence of attacks, and the black circles show the timing of the confirmation of AQP4‐IgG positivity. Abbreviations: APS, area postrema syndrome; AQP4‐IgG, anti‐aquaporin‐4 antibodies; CF, counting fingers; HM, hand motion; IVMP, intravenous methylprednisolone pulse therapy; LP, light perception; ON, optic neuritis; PE, plasma exchange; PSL, prednisolone; VA, visual acuity

The clinical courses of the three cases are shown in Figure 1.

**FIGURE 2:**
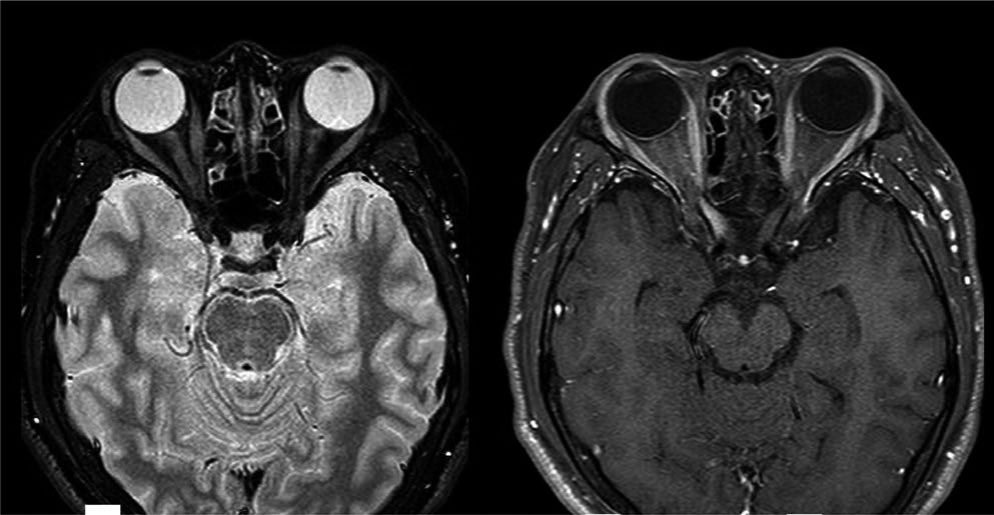
Optic MRI on Day 14 after trauma in Case 1. Optic MRI in the STIR sequence (left) and contrast‐enhanced fat‐suppressed T1‐weighted (right) are shown

Case 1 was a 16‐year‐old male patient who experienced trauma to his right eye. He previously had a neurological episode of intractable hiccup at 13 years of age, and 2 months before the minor trauma he experienced urinary retention that required urethral catheterization. He retrospectively recalled a strange feeling in his right eye when he put on his contact lens two days before the trauma. He received trauma to his right eye when he accidentally fell down on his right side while snowboarding. He noticed blurred vision in his right eye, which gradually exacerbated over the following 2 weeks. The nadir corrected visual acuity (VA) in his right eye at day 14 was 0.03 on the decimal scale. Fundus examination revealed normal results. Contrast‐enhanced MRI revealed optic nerve swelling and contrast enhancement in the right optic nerve, compatible with a diagnosis of ON (Figure 2). The patient was rapidly treated with one cycle of high‐dose intravenous methylprednisolone pulse therapy (IVMP) and three cycles of plasma exchange (PE), and he recovered his full visual acuity. At present, the patient is on oral prednisolone (PSL) therapy at a daily dose of 10 mg, and he has had no relapse since the ON episode related to the trauma.

Case 2 was a 44‐year‐old female patient who experienced trauma to her right eye. She had no history of neurological episodes before the trauma. While asleep, her family member's hand accidentally hit her right eye while turning over in bed. When she woke up the next morning, she found that she had no vision in her right eye. She visited an ophthalmologist who found that her corrected VA in the right eye was light perception (LP). Fundus examination revealed normal results. She was diagnosed with traumatic optic neuropathy and was not treated with any medication. Her visual acuity did not recover. When she was 45 years old she had two neurological episodes, left ON and cervical acute myelitis, and both were swiftly treated with IVMP. When she was 52 years old, serum AQP4‐IgG was checked for the first time and she tested positive, resulting in a diagnosis of AQP4‐IgG‐positive NMOSD. Oral prednisolone and azathioprine combination therapy was started to prevent relapse. At present, she is 65 years old, with the VA of counting fingers (CF) in the right eye, and 0.1 in the left eye on a decimal scale.

Case 3 was a 34‐year‐old woman who experienced trauma with a tennis ball to her right eye. About three years before this happened, she had another episode of trauma to her right eye with a hard baseball. She experienced severe ocular hyperemia and epiphora after the first trauma with a baseball, but her visual acuity did not decrease then. When she was 34 years old, a tennis ball hit her right eye on the first bounce. Because the hit was weak and she felt no abnormality, she continued to play tennis. Two days after the minor trauma, she had a strange feverish and swollen feeling in her right eye, and she visited an ophthalmologist. Her corrected VA in the right eye was 0.6, which was lower than that of the healthy side with 1.2. Fundus examination revealed normal results. Head and orbital CT images revealed no structural damage. She was treated with low‐dose oral PSL, but her VA gradually dropped with a nadir VA of 0.3, which was reached around Day 60. After this episode, she was started on low‐dose oral PSL for relapse prevention, but she again experienced attacks at 39, 43, 44, and 50 years of age. When she was 52 years old, serum AQP4‐IgG was checked for the first time and she tested positive. Based on this result, oral azathioprine was added to the PSL, but she still experienced attacks at 59 and 64 years. At present, she is 65 years old, and her corrected VA is CF in the right eye and 1.0 (decimal scale) in the left eye.

## 4 DISCUSSION

In this report, three patients with AQP4‐IgG‐positive NMOSD experienced acute ON after a minor traumatic injury to their eyes without fundus abnormality or orbital floor fracture. All three patients had recurrent neurological attacks of acute ON, acute myelitis, or APS, before or after the traumatic episodes, which is compatible with the clinical course of AQP4‐IgG‐positive NMOSD. Usually, visual disturbances based on traumatic injury do not manifest as inflammatory ON. Thus, such a condition is called traumatic optic neuropathy and not neuritis. Most cases of traumatic optic neuropathy require mechanical shearing of optic nerves or impaired microcirculation in the optic nerve canals (Samardzic et al., 2012; Vorwerk et al., 2004). As the situations of the injury, high‐energy head or orbital trauma (e.g., traffic accident, fall trauma, and violence) are popular with or without bone fractures (Kumaran et al., 2015; Lee et al., 2010). This results in macroscopic physical damage to the surrounding periorbital structures or to the optic nerve itself. Meanwhile, all three presented cases received minor trauma without high‐energy impact or macroscopic structural damage (e.g., orbital floor fractures or skull base fracture) or fundus abnormalities (e.g., retinal detachment). If we suppose that the minor trauma without macroscopic structural damage triggered the inflammatory acute ON in them, two hypothetical mechanisms could be considered. First, the patients had some predisposing factors ahead of the traumatic injury, such as the presence of serum AQP4‐IgG, and the trauma triggered the advent of acute ON. Serum AQP4‐IgG has been reported to be possibly present before the clinical onset of NMOSD (Nishiyama et al., 2009). The second theory is that in patients without serum AQP4‐IgG, the trauma either caused or increased the exposure of AQP4 protein to the lymphocytes circulating in the blood or cerebrospinal fluid, which activated humoral immunity and, subsequently, the differentiation of AQP4‐specific plasmablasts. However, in case of the differentiation of AQP4‐specific plasmablasts, acute ON episodes would occur several days after the trauma. In light of these findings, we believe that some predisposing factors such as the presence of serum AQP4‐IgG before the traumatic injury in our three patients have played a role in the onset of acute ON; even minor traumas that usually cause no severe problems in healthy subjects might have triggered acute ON in these cases due to the predisposing factors.

The possible association between physical trauma and the subsequent diagnosis of inflammatory demyelinating diseases in the central nervous system has been discussed previously, especially in multiple sclerosis (Goldacre et al., 2006; Kang & Lin, 2012; Poser, 2000). Impaired blood–brain barrier permeability in the traumatically damaged nervous system is thought to play a role in the formation of inflammatory lesions in the central nervous system. Systematic reviews and meta‐analyses involving case–control studies suggested a significant association between head trauma and a subsequent diagnosis of multiple sclerosis (Lunny et al., 2014; Warren et al., 2013). However, meta‐analyses that assessed only cohort studies reported no such association. In total, the causal or triggering association between physical trauma and the subsequent advent of inflammatory lesions in the central nervous system has not been fully established at present. Further investigation of relevant cases is needed to better understand the association between the two events.

The post‐traumatic ON episodes noted in the three cases are clinically significant as it suggests the following. Trauma to the head and eyes could have severe consequences in people with serum AQP4‐IgG and must therefore be avoided. In case of acute ON after minor trauma in subjects with unknown presence of serum AQP4‐IgG, it would be better to screen for the presence of serum AQP4‐IgG for making a correct diagnosis and deciding appropriate therapeutic strategy. Lastly, regardless of the presence of traumatic episodes ahead of the acute ON, timely administration of high‐dose intravenous steroid pulse therapy is needed. Because the expected visual prognosis is generally worse in AQP4‐IgG‐positive NMOSD than in other multiple sclerosis and related disorders (Akaishi et al., 2016; Srikajon et al., 2018), active acute treatments with or without adjunctive plasma exchange are required in patients with ON related to AQP4‐IgG. Certainly, the use of steroids in the treatment of traumatic optic neuropathy is not evident (Yu‐Wai‐Man & Griffiths, 2011), but it would be better to discriminate the two diseases of traumatic optic neuropathy and AQP4‐IgG‐related ON triggered by minor trauma. When there is no confirmed macroscopic structural damage that can explain the visual impairment, the positivity of serum AQP4‐IgG would be better to be checked before making a definite diagnosis of traumatic optic neuropathy and deciding not to use the steroids.

As a limitation of this study, Case 2 and Case 3 were checked for their serum AQP4‐IgG levels more than 10 years after the occurrence of traumatic injury. This was because the existence of AQP4‐IgG in some patients with neuromyelitis optica was not yet known at the time of their injury (Lennon et al., 2004). However, the clinical courses of these two cases before the confirmation of serum AQP4‐IgG are compatible with those of AQP4‐IgG‐positive NMOSD, suggesting that they already possessed AQP4‐IgG at the occurrence of their traumatic injuries.

In conclusion, minor trauma to the eyes with or without major fundus or periorbital damage may trigger inflammatory acute ON in patients with predisposing risk factors such as the positivity of serum AQP4‐IgG. Patients with AQP4‐IgG‐positive NMOSD need to be extra careful to avoid head and eye trauma that may trigger acute ON. Once the patient is diagnosed with post‐traumatic inflammatory ON related to serum AQP4‐IgG, rapid initiation of intravenous steroid pulse therapy is necessary to minimize severe irreversible sequelae.

## CONFLICT OF INTEREST

None declared.

## AUTHOR CONTRIBUTION

T Akaishi, N Himori, T Takeshita, and I Nakashima designed the study. T Akaishi, N Himori, T Takeshita, K Fujihara, and T Misu collected and prepared the patient information. T Takahashi performed antibody titrations. T Akaishi, T Takeshita, J Fujimori, and I Nakashima interpreted the data. T Akaishi, N Himori, T Takeshita, and I Nakashima drafted the manuscript. All of the authors critically reviewed and revised the manuscript. K Fujihara, T Ishii, M Aoki, T Nakazawa, and I Nakashima supervised the study.

## Data Availability

The anonymized data that support the findings of this study are available from the corresponding author upon reasonable request.
